# The plasma peptides of breast versus ovarian cancer

**DOI:** 10.1186/s12014-019-9262-0

**Published:** 2019-12-23

**Authors:** Jaimie Dufresne, Pete Bowden, Thanusi Thavarajah, Angelique Florentinus-Mefailoski, Zhuo Zhen Chen, Monika Tucholska, Tenzin Norzin, Margaret Truc Ho, Morla Phan, Nargiz Mohamed, Amir Ravandi, Eric Stanton, Arthur S. Slutsky, Claudia C. dos Santos, Alexander Romaschin, John C. Marshall, Christina Addison, Shawn Malone, Daren Heyland, Philip Scheltens, Joep Killestein, Charlotte Teunissen, Eleftherios P. Diamandis, K. W. M. Siu, John G. Marshall

**Affiliations:** 1 Ryerson Analytical Biochemistry Laboratory (RABL), Department of Chemistry and Biology, Faculty of Science, Ryerson University, 350 Victoria St., Toronto, ON Canada; 2 Institute of Cardiovascular Sciences, St. Boniface Hospital Research Center, University of Manitoba, Winnipeg, Canada; 3 Division of Cardiology, Department of Medicine, McMaster University, Hamilton, Canada; 4 St. Michael’s Hospital, Keenan Chair in Medicine, Professor of Medicine, Surgery & Biomedical Engineering, University of Toronto, Toronto, Canada; 5 St. Michael’s Hospital, Keenan Research Centre for Biomedical Science, Toronto, Canada; 6 Program for Cancer Therapeutics, Ottawa Hospital Research Institute, Ottawa, Canada; 7 Clinical Evaluation Research Unit, Kingston General Hospital, Kingston, Canada; 8 Alzheimer Center, Dept of Neurology, Amsterdam University Medical Centers, Vrije Universiteit, Amsterdam Neuroscience, Amsterdam, The Netherlands; 9 MS Center, Dept of Neurology, Amsterdam University Medical Centers, Vrije Universiteit, Amsterdam Neuroscience, Amsterdam, The Netherlands; 10 Neurochemistry Lab and Biobank, Dept of Clinical Chemsitry, Amsterdam University Medical Centers, Vrije Universiteit, Amsterdam Neuroscience, Amsterdam, The Netherlands; 11 Mount Sinai Hospital Research Institute, University of Toronto, Toronto, Canada; 12 University of Windsor, Windsor, Canada; 13 International Biobank of Luxembourg (IBBL), Luxembourg Institute of Health (formerly CRP Sante Luxembourg), Strassen, Luxembourg

**Keywords:** Human EDTA plasma, Organic extraction, Nano chromatography, Electrospray ionization tandem mass spectrometry, LC–ESI–MS/MS, Linear quadrupole ion trap, Discovery of variation, Breast cancer, Random and independent sampling, Chi Square test and ANOVA, SQL SERVER and R

## Abstract

**Background:**

There is a need to demonstrate a proof of principle that proteomics has the capacity to analyze plasma from breast cancer versus other diseases and controls in a multisite clinical trial design. The peptides or proteins that show a high observation frequency, and/or precursor intensity, specific to breast cancer plasma might be discovered by comparison to other diseases and matched controls. The endogenous tryptic peptides of breast cancer plasma were compared to ovarian cancer, female normal, sepsis, heart attack, Alzheimer’s and multiple sclerosis along with the institution-matched normal and control samples collected directly onto ice.

**Methods:**

Endogenous tryptic peptides were extracted from individual breast cancer and control EDTA plasma samples in a step gradient of acetonitrile, and collected over preparative C18 for LC–ESI–MS/MS with a set of LTQ XL linear quadrupole ion traps working together in parallel to randomly and independently sample clinical populations. The MS/MS spectra were fit to fully tryptic peptides or phosphopeptides within proteins using the X!TANDEM algorithm. The protein observation frequency was counted using the SEQUEST algorithm after selecting the single best charge state and peptide sequence for each MS/MS spectra. The observation frequency was subsequently tested by Chi Square analysis. The log_10_ precursor intensity was compared by ANOVA in the R statistical system.

**Results:**

Peptides and/or phosphopeptides of common plasma proteins such as APOE, C4A, C4B, C3, APOA1, APOC2, APOC4, ITIH3 and ITIH4 showed increased observation frequency and/or precursor intensity in breast cancer. Many cellular proteins also showed large changes in frequency by Chi Square (χ^2^ > 100, p < 0.0001) in the breast cancer samples such as CPEB1, LTBP4, HIF-1A, IGHE, RAB44, NEFM, C19orf82, SLC35B1, 1D12A, C8orf34, HIF1A, OCLN, EYA1, HLA-DRB1, LARS, PTPDC1, WWC1, ZNF562, PTMA, MGAT1, NDUFA1, NOGOC, OR1E1, OR1E2, CFI, HSA12, GCSH, ELTD1, TBX15, NR2C2, FLJ00045, PDLIM1, GALNT9, ASH2L, PPFIBP1, LRRC4B, SLCO3A1, BHMT2, CS, FAM188B2, LGALS7, SAT2, SFRS8, SLC22A12, WNT9B, SLC2A4, ZNF101, WT1, CCDC47, ERLIN1, SPFH1, EID2, THOC1, DDX47, MREG, PTPRE, EMILIN1, DKFZp779G1236 and MAP3K8 among others. The protein gene symbols with large Chi Square values were significantly enriched in proteins that showed a complex set of previously established functional and structural relationships by STRING analysis. An increase in mean precursor intensity of peptides was observed for QSER1 as well as SLC35B1, IQCJ-SCHIP1, MREG, BHMT2, LGALS7, THOC1, ANXA4, DHDDS, SAT2, PTMA and FYCO1 among others. In contrast, the QSER1 peptide QPKVKAEPPPK was apparently specific to ovarian cancer.

**Conclusion:**

There was striking agreement between the breast cancer plasma peptides and proteins discovered by LC–ESI–MS/MS with previous biomarkers from tumors, cells lines or body fluids by genetic or biochemical methods. The results indicate that variation in plasma peptides from breast cancer versus ovarian cancer may be directly discovered by LC–ESI–MS/MS that will be a powerful tool for clinical research. It may be possible to use a battery of sensitive and robust linear quadrupole ion traps for random and independent sampling of plasma from a multisite clinical trial.

## Introduction

### Blood peptides

The endogenous peptides of human serum and plasma were first detected by highly sensitive MALDI [1–3]. The MALDI “patterns” formed by the ex vivo degradation of the major peptides of human blood fluids have been compared using complex multivariate approaches [4–6]. It was suggested that pattern analysis of endo-proteinases or exo-peptidases would permit the diagnosis of cancer [7, 8]. However, there was no evidence that multivariate pattern analysis of the peptides or exo-peptidase activity will serve as a valid diagnostic [9]. Multivariate pattern analysis is prone to over-interpretation of laboratory or clinical experiments [10, 11]. Univariate ANOVA of the main feature(s) provided about the same statistical power as multivariate analysis [12]. The endogenous peptides of human blood were first identified by MS/MS fragmentation using MALDI-Qq-TOF and LC–ESI–MS/MS with an ion trap mass spectrometer, that showed excellent agreement with exogenous digestions, and the intensity values compared by ANOVA [12, 13]. Random and independent sampling of the endogenous tryptic peptides from clinical plasma samples revealed individual peptides or proteins that show significant variation by standard statistical methods such as the Chi Square test and ANOVA [12, 14–18]. Pre-analytical variation was exhaustively studied between fresh EDTA plasma samples on ice versus plasma samples degraded for various lengths of time to control for differences in sample handling and storage. The observation frequency of peptides from many proteins may increase by on average twofold after incubation at room temperature [17–19] and indicates that Complement C3 and C4B vary with time of incubation ex vivo [17, 18] in agreement with previous results [12].

### Sample preparation

The sensitive analysis of human blood fluids by LC–ESI–MS/MS is dependent on effective fractionation strategies, such as partition chromatography or organic extraction, to relieve suppression and competition for ionization, resulting in high signal to noise ratios and thus low error rates of identification and quantification [20]. Without step wise sample partition only a few high abundance proteins may be observed from blood fluid [13, 21, 22]. In contrast, with sufficient sample preparation, low abundance proteins of ≤ 1 ng/ml could be detected and quantified in blood samples by mass spectrometry [22, 23]. Simple and single-use, i.e. disposable, preparative and analytical separation apparatus permits the identification and quantification of blood peptides and proteins with no possibility of cross contamination between patients that guarantees sampling is statistically independent [12, 13, 17, 22, 23]. Previously, the use of precipitation and selective extraction of the pellet [23–26] was shown to be superior to precipitation and analysis of the ACN supernatant [27], ultra-filtration, [28] albumin depletion chromatography [29] or C18 partition chromatography alone [13]. Precipitating all of the polypeptides with 90% ACN followed by step-wise extraction of the peptides with mixtures of organic solvent and water was the optimal method to sensitively detect peptides from blood [21]. Here a step gradient of acetonitrile/water to extract 200 µl of EDTA plasma for analysis by LC–ESI–MS/MS showed a high signal to noise ratio [21] and resulted in the confident identification of tryptic peptides [17] from breast cancer versus normal control samples.

### Computation and statistics

Partition of each clinical sample into multiple sub-fractions, that each must be randomly and independently sampled by analytical C18 LC–ESI–MS/MS provides sensitivity [21] but also creates a large computational challenge. Previously the 32-bit computer power was lacking to identify and compare all the peptides and protein from thousands of LC–ESI–MS/MS recordings in a large multisite clinical experiment [30]. Here we show the MS/MS spectra from random and independent sampling of peptides from 1508 LC–ESI–MS/MS experiments from multiple clinical treatments and sites may be fit to peptides using a 64 bit server and then the observation frequency and precursor intensity compared across treatments using SQL SERVER/R that shows excellent data compression and relation [14, 17]. The protein p-values and FDR q-values were computed from organic extraction or chromatography of blood fluid and the peptide-to-protein distribution of the precursor ions of greater than ~ 10,000 (E4) counts were compared to a null (i.e. known false positive) model of noise or computer generated random MS/MS spectra [15, 17, 31–34]. Peptides may be identified from the fit of MS/MS spectra to peptide sequences [35] that permits the accurate estimate of the type I error rate (*p* value) of protein identification that may be corrected by the method Benjamini and Hochberg [36] to yield the FDR (q-value) [17, 21, 31]. The peptide fits may be filtered from redundant results to the single best fit of the peptide sequence and charge state using a complex key in SQL Server [17, 31, 37, 38]. Simulations using random or noise MS/MS spectra distributions may be used to control the type I error of experimental MS/MS spectra correlations to tryptic peptides [15–17, 31–34, 37]. The peptide and protein observation counts (frequency) may be analyzed using classical statistic methods such as Chi Square analysis [33, 39]. Log_10_ transformation of precursor intensity yields a normal distribution that permits comparison of peptide and proteins expression levels by ANOVA [15, 16]. The SQL Server system permits the direct interrogation of the related data by the open source R statistical system without proteomic-specific software packages. Here the use of SQL/R has permitted the detailed statistical analysis of randomly and independently sampled LC–ESI–MS/MS data from multiple hospitals in parallel that would be requisite for a multisite clinical trial [37, 39].

### Cancer proteins in blood fluids

Markers of breast cancer [40] have been examined from nano vesicles [41] that may mediate tumor invasion [42], in proximal fluid [43, 44] or from serum or plasma [45–47]. Many non-specific, i.e. “common distress” or “acute phase” proteins have been detected to increase by the analysis of blood fluids such as amyloids, haptoglobin, alpha 1 antitrypsin, clusterin, apolipoproteins, complement components, heat shock proteins, fibrinogens, hemopexin, alpha 2 macroglobulin and others that may be of limited diagnostic value [20, 48, 49]. There is good evidence that cellular proteins may exist in circulation, and even form supramolecular complexes with other molecules, in the blood [50]. Proteins and nucleic acids may be packaged in exosomes that are challenging to isolate [51, 52] and it appears that cellular proteins may be secreted into circulation [50, 53, 54]. Here, the combination of step wise organic partition [21], random and independent sampling by nano electrospray LC–ESI–MS/MS [17], and 64 bit computation with SQL SERVER/R [14] permitted the sensitive detection of peptides and/or phosphopeptides from human plasma. The variation in endogenous peptides within parent protein chains in computed complexes from breast cancer patients versus ovarian cancer and other disease and normal plasma were compared by the classical statistical approaches of the Chi Square test followed by univariate ANOVA [12, 15, 16].

## Materials and methods

### Materials

Anonymous human EDTA plasma with no identifying information from multiple disease and control populations were transported frozen and stored in a − 80 ºC freezer. Breast cancer vs ovarian cancer disease and matched normal female human EDTA plasma was obtained from the Ontario Tumor Bank of the Ontario Institute of Cancer Research, Toronto Ontario. Additional controls of heart attack (venous and arterial) and normal pre-operative orthopedic samples were from St. Joseph’s Hospital of McMaster University. ICU-Sepsis and ICU-Alone were obtained from St. Michael’s Hospital Toronto. Multiple sclerosis, Alzheimer’s dementia and normal controls were from Amsterdam University Medical Center, Vrije Universiteit Amsterdam. In addition, EDTA plasma samples collected onto ice as a baseline degradation controls were obtained from IBBL Luxembourg and stored freeze dried. The anonymous plasma samples with no identifying information from the multiple clinical locations were analyzed under the Ryerson Research Ethics Board Protocol REB 2015-207. C18 zip tips were obtained from Millipore (Bedford, MA), C18 HPLC resin was from Agilent (Zorbax 300 SB-C18 5-micron). Solvents were obtained from Caledon Laboratories (Georgetown, Ontario, Canada). All other salts and reagents were obtained from Sigma-Aldrich-Fluka (St Louis, MO) except where indicated. The level of replication in the LC–ESI–MS-MS experiments was typically between 9 and 26 independent patient plasma samples for each disease and control.

### Sample preparation

Human EDTA plasma samples (200 μl) were precipitated with 9 volumes of acetonitrile (90% ACN) [23], followed by the selective extraction of the pellet using a step gradient to achieve selectivity across sub-fractions and thus greater sensitivity [21]. Disposable plastic 2 ml sample tubes and plastic pipette tips were used to handle samples. The acetonitrile suspension was separated with a centrifuge at 12,000 RCF for 5 min. The acetonitrile supernatant, that contains few peptides, was collected, transferred to a fresh sample tube and dried in a rotary lyophilizer. The organic precipitate (pellet) that contains a much larger total amount of endogenous polypeptides [23] was manually re-suspended using a step gradient of increasing water content to yield 10 fractions from those soluble in 90% ACN to 10% ACN, followed by 100% H_2_O, and then 5% formic acid [21]. The step-wise extracts were clarified with a centrifuge at 12,000 RCF for 5 min. The extracted sample fractions were dried under vacuum in a rotary lyophillizer and stored at − 80 °C for subsequent analysis.

### Preparative C18 chromatography

The peptides of EDTA plasma were precipitated in ACN, extracted from the pellet in a step-gradient with increasing water, dried and then collected over C18 preparative partition chromatography. Preparative C18 separation provided the best results for peptide and phosphopeptide analysis in a “blind” analysis [55]. Solid phase extraction with C18 for LC–ESI–MS/MS was performed as previously described [12, 13, 22–24]. The C18 chromatography resin (Zip Tip) was wet with 65% acetonitrile and 5% formic acid before equilibration in water with 5% formic acid. The plasma extract was dissolved in 200 μl of 5% formic acid in water for C18 binding. The resin was washed with at least five volumes of the binding buffer. The resin was eluted with ≥ 3 column volumes of 65% acetonitrile (2 µl) in 5% formic acid. In order to avoid cross-contamination the preparative C18 resin was discarded after a single use.

### LC–ESI–MS/MS

In order to entirely prevent any possibility of cross contamination, a new disposable nano analytical HPLC column and nano emitter was fabricated for recording each patient sample-fraction set. The ion traps were cleaned and tested for sensitivity with angiotensin and glu fibrinogen prior to recordings. The new column was conditioned and quality controlled with a mixture of three non-human protein standards [32] using a digest of Bovine Cytochrome C, Yeast alcohol dehydrogenase (ADH) and Rabbit Glycogen Phosphorylase B to confirm the sensitivity and mass accuracy of the system prior to each patient sample set. The statistical validity of the LTQ XL (Thermo Electron Corporation, Waltham, MA, USA) linear quadrupole ion trap for LC–ESI–MS/MS of human plasma [21] was in agreement with the results from the 3D Paul ion trap [15, 32–34]. The stepwise extractions were collected and desalted over C18 preparative micro columns, eluted in 2 µl of 65% ACN and 5% formic acid, diluted tenfold with 5% formic acid in water and immediately loaded manually into a 20 μl metal sample loop before injecting onto the analytical column via a Rhodynne injector. Endogenous peptide samples were analyzed over a discontinuous gradient generated at a flow rate of ~ 10 μl per minute with an Agilent 1100 series capillary pump and split upstream of the injector during recording to about ~ 200 nl per minute. The separation was performed with a C18 (150 mm × 0.15 mm) fritted capillary column. The acetonitrile profile was started at 5%, ramped to 12% after 5 min and then increased to 65% over ~ 90 min, remained at 65% for 5 min, decreased to 50% for 15 min and then declined to a final proportion of 5% prior to injection of the next step fraction from the same patient. The nano HPLC effluent was analyzed by ESI ionization with detection by MS and fragmentation by MS/MS with a linear quadrupole ion trap [56]. The device was set to collect the precursors for up to 200 ms prior to MS/MS fragmentation with up to four fragmentations per precursor ion that were averaged. Individual, independent samples from disease, normal and ice cold control were precipitated, fractionated over a step gradient and collected over C18 for manual injection.

### Correlation analysis

Correlation analysis of ion trap data was performed using a goodness of fit test by X!TANDEM [35] and by cross-correlation using SEQUEST [57] on separate servers to match tandem mass spectra to peptide sequences from the Homo sapiens RefSeq, Ensembl, SwissProt, including hypothetical proteins XP or Genomic loci [13, 14, 58]. Endogenous peptides with precursors greater than 10,000 (E4) arbitrary counts were searched only as fully tryptic peptides (TRYP) and/or phosphopeptides (TYRP STYP) and compared in SQL Server/R. The X!TANDEM default ion trap data settings of ± 3 m/z from precursors peptides considered from 300 to 2000 m/z with a tolerance of 0.5 Da error in the fragments were used [15, 22, 33–35, 59]. The best fit peptide of the MS/MS spectra to fully tryptic and/or phospho-tryptic peptides at charge states of + 2 versus + 3 were accepted with additional acetylation, or oxidation of methionine and with possible loss of water or ammonia. The resulting accession numbers, actual and estimated masses, correlated peptide sequences, peptide and protein scores, resulting protein sequences and other associated data were captured and assembled together in an SQL Server relational database [14].

### Data sampling, sorting, transformation and visualization

Each disease and normal treatment was represented by 9 to 26 independent patient samples that were resolved into 10 organic/water sub-fractions resulting in 90 to 260 sub-samples per treatment for a total of 1508 LC–ESI–MS/MS experiments that were archived together in SQL Server for statistical analysis [37, 39]. The linear quadrupole ion trap provided the precursor ion intensity values and the peptide fragment MS/MS spectra. The peptides and proteins were identified from MS/MS spectra by X!TANDEM and the observation frequency was counted by the SEQUEST algorithm. The large number of redundant correlations to each MS/MS at different charge states or to different peptides sequences may be a source of type I error that can be filtered out by a complex key or hashtag in SQL Server to ensure that each MS/MS spectra is only fit to one peptide and charge state. The MS and MS/MS spectra together with the results of the X!TANDEM and SEQUEST algorithms were parsed into an SQL Server database and filtered [14] before statistical and graphical analysis with the generic R data system [14–16, 32, 58]. The sum of the MS/MS spectra collected in breast versus ovarian cancer were summed to correct the observation frequency using Eq. 1 and the χ^2^ p-values converted to FDR q-values by the method of Benjamini and Hochberg [36]:

1 $$({\text{Breast}} - {\text{Ovarian}})^{2} / ({\text{Ovarian}} + 1)$$

Correction by sum correlations yielded similar results (not shown). The precursor intensity data for MS/MS spectra were log_10_ transformed, tested for normality and analyzed across institution/study and diseases verses controls by means, standard errors and ANOVA [15, 16, 32]. The entirely independent analysis of the precursor intensity using the rigorous ANOVA with Tukey–Kramer HSD test versus multiple controls was achieved using a 64 bit R server.

## Results

Partition of plasma samples using differential solubility in organic/water mixtures combined with random and independent sampling by LC–ESI–MS/MS detected peptides from proteins that were more frequently observed and/or showed greater intensity in breast versus ovarian cancer. Here four independent lines of evidence, Chi Square analysis of observation frequency, previously established structural/functional relationships from STRING, ANOVA analysis of peptide intensity, and agreement with the previous genetic or biochemical experiments, all indicated that there was significant variation in the peptides of breast cancer patients compared to ovarian cancer and other diseases or normal plasma samples.

### LC–ESI–MS/MS

The pool of endogenous tryptic (TRYP) and/or tryptic phosphopeptides (TRYP STYP) were randomly and independently sampled without replacement by liquid chromatography, nano electrospray ionization and tandem mass spectrometry (LC–ESI–MS/MS) [17] from breast vs ovarian cancer, or female normal, other disease and normal plasma, and ice cold controls to serve as a baseline [18, 19]. Some 15,968,550 MS/MS spectra ≥ E4 intensity counts were correlated by the SEQUEST and X!TANDEM algorithms that resulted in a total of 19,197,152 redundant MS/MS spectra to peptide in protein matches. The redundant correlations from SEQUEST were filtered to retain only the best fit by charge state and peptide sequence in SQL Server to entirely avoid re-use of the same MS/MS spectra [17, 31, 37, 39]. The filtered results were then analyzed by the generic R statistical system in a matrix of disease and controls that reveals the set of blood peptides and proteins specific to each disease state. The statistical validity of the extraction and sampling system were previously established by computation of protein (gene symbol) p-values and FDR corrected q-values by the method of Benjamini and Hochberg [36] and frequency comparison to false positive noise or random spectra [17, 21].

### Frequency correction

**Fig. 1:**
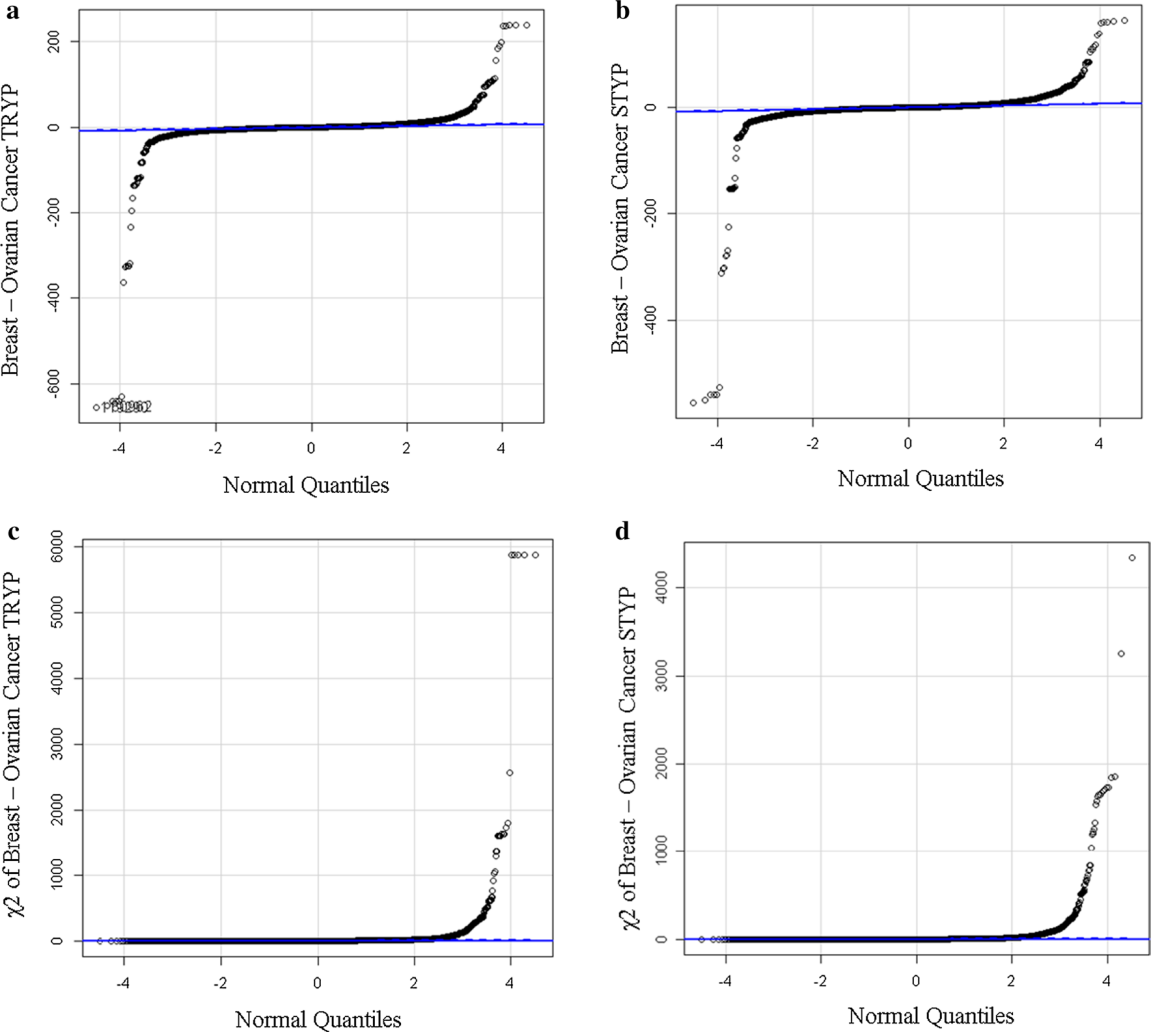
Quantile plots of the corrected difference and Chi Square values of the Breast Cancer versus Ovarian Cancer results after frequency correction. The difference of breast cancer (n ≥ 9) versus ovarian cancer (n ≥ 9) using the quantile plot that tended to zero (see quantile line). Similar results were obtained by comparison to breast cancer or other controls (not shown). Plots: ** a** quantile plot of the observation frequency of tryptic peptides from breast cancer–ovarian cancer; ** b** χ^2^ plot of the observation frequency of tryptic peptides from breast cancer–ovarian cancer tryptic peptides; **c** quantile plot of the observation frequency of tryptic STYP peptides from breast cancer–ovarian cancer; **d** χ^2^ plot of the observation frequency of tryptic STYP peptides from breast cancer–ovarian cancer tryptic peptides

A total of 455,426 MS/MS ≥ E4 counts were collected from breast cancer samples and 498,616 MS/MS ≥ E4 counts were collected from ovarian cancer plasma and these sums were used to correct observation frequency. A small subset of proteins show large increases or decreases in observation frequency between breast versus ovarian cancer resulting in large Chi Square values (Fig. 1). Similar results were obtained from comparison to female normal (not shown).

### Comparison of breast cancer to ovarian cancer by Chi square analysis

A set of ~ 500 gene symbols showed Chi Square (χ^2^) values ≥ 15 between breast cancer versus ovarian cancer. Specific peptides and/or phosphopeptides from cellular proteins, membrane proteins, nucleic acid binding proteins, signaling factors, metabolic enzymes and others, including uncharacterized proteins, showed significantly greater observation frequency in breast cancer. In agreement with the literature, peptides from many established plasma proteins including acute phase or common distress proteins such as APOE, C4A, C4B, C4B2, C3, CFI, APOA1, APOC2, APOC4-APOC2, IGHE, ITIH3, and ITIH4 [60, 61] were observed to vary between cancer and control samples. The Chi Square analysis showed some proteins with χ^2^ values that were apparently too large (χ^2^ ≥ 60, p < 0.0001, d.f. 1) to all have resulted from random sampling error. Many cellular proteins also showed large changes in frequency by Chi Square (χ^2^ > 100, p < 0.0001) in the breast cancer samples such as CPEB1, LTBP4, HIF-1A, IGHE, RAB44, NEFM, C19orf82, SLC35B1, 1D12A, C8orf34, HIF1A, OCLN, EYA1, HLA-DRB1, LARS, PTPDC1, WWC1, ZNF562, PTMA, MGAT1, NDUFA1, NOGOC, OR1E1, OR1E2, CFI, HSA12, GCSH, ELTD1, TBX15, NR2C2, FLJ00045, PDLIM1 GALNT9, ASH2L, PPFIBP1, LRRC4B, SLCO3A1, BHMT2, CS, FAM188B2, LGALS7, SAT2, SFRS8, SLC22A12, WNT9B, SLC2A4, ZNF101, WT1, CCDC47, ERLIN1, SPFH1, EID2, THOC1, DDX47, MREG, PTPRE, EMILIN1, DKFZp779G1236 and MAP3K8 among others (Table 1). The full list of Chi Square results are found in the Additional file 1: Table S1.

**Table 1:** Breast cancer specific proteins detected by fully tryptic peptides and/or fully tryptic phosphopeptides (STYP) that show a Chi Square (χ^2^) value of ≥ 200. N is the number of protein accessions per Gene Symbol

Tryptic Gene_Symbol			Tryptic STYP		
Gene Symbol	Mean X2	n	Gene Symbol	Mean X2	n
CPEB1	3632.919337	8	LTBP4	4340.217566	1
LTBP4	2560.471517	1	C19orf82	3256.703566	1
HIF-1A	1640.975019	1	PMEPA1	1849.257201	1
C4A	1626.866928	2	C4A	1703.128264	2
C4B	1626.866928	2	HIF-1A	1668.954624	1
C4B_2	1612.006355	1	C4B_2	1648.102936	1
C3	757.057969	2	C4B	1637.227896	2
IGHE	656.105042	1	CA7	1582.270693	1
RAB44	656.105042	1	PCDHGA5	1462.852842	2
NEFM	652.140957	5	C8orf34	1189.441768	5
C19orf82	613.883173	1	C3	835.343196	2
SLC35B1	479.46677	1	KNOP1	822.636731	3
C8orf34	460.113072	5	AMMECR1L	794.024811	5
1D12A	432.71876	1	HMMR	699.705336	1
HIF1A	352.516679	3	HTR3B	670.791156	1
OCLN	341.835514	3	PCDHJ	611.647195	1
APOE	336.148697	3	ZFAND1	522.966422	2
PTPDC1	316.183187	2	PPID	522.527735	1
EYA1	306.858733	1	OXER1	509.701516	1
HLA-DRB1	306.858733	1	DCHS2	507.103436	1
WWC1	294.679057	9	RAB44	449.029189	1
ZNF562	273.551291	13	NUP50	431.635555	4
CFI	251.996191	7	HLA-DRB1	417.238656	1
MGAT1	241.814491	1	PCED1A	375.630369	4
NDUFA1	241.814491	1	HIF1A	304.82744	3
NOGOC	241.814491	1	CHMP5	297.080368	2
OR1E1	241.814491	1	HMP19	289.436434	5
OR1E2	241.814491	1	LOC102723665	286.501857	1
PTMA	234.938717	1	CYC1	260.817537	2
HSA12	218.336655	1	GCSH	260.051794	1
ELTD1	206.644334	1	CNBP	259.243457	7
GCSH	202.57471	1	SMIM12	256.548507	1

### Pathway and gene ontology analysis using the STRING algorithm

**Fig. 2:**
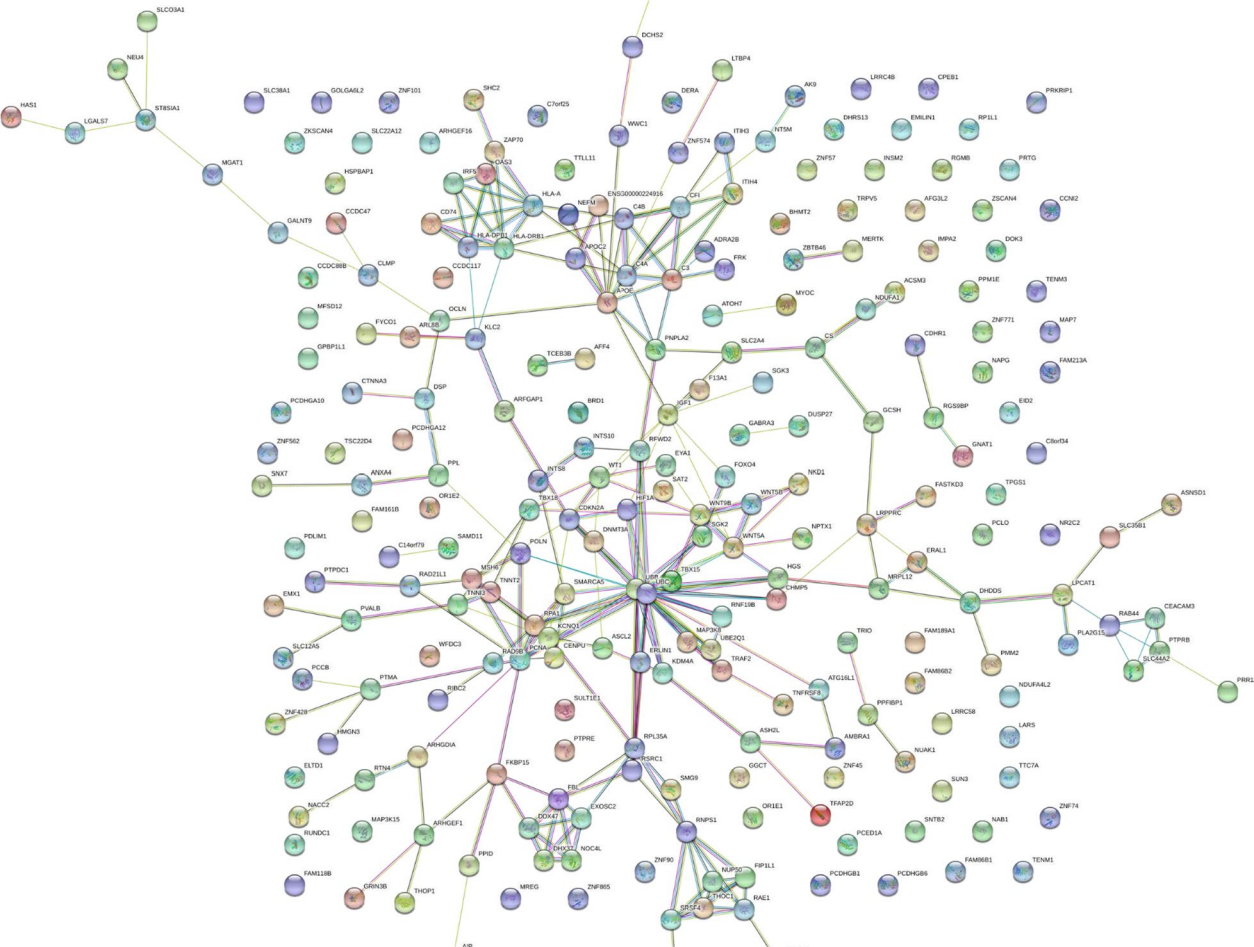
The breast cancer STRING network where Chi Square χ^2^ ≥ 15 from fully tryptic peptides. Breast cancer tryptic peptide frequency difference greater than 15 and χ^2^ value greater than 15 at degrees of freedom of 1 (p < 0.0001). Network Stats: number of nodes, 173; number of edges, 260; average node degree, 3.01; avg. local clustering coefficient, 0.378; expected number of edges, 206; PPI enrichment p-value, 0.000175

**Fig. 3:**
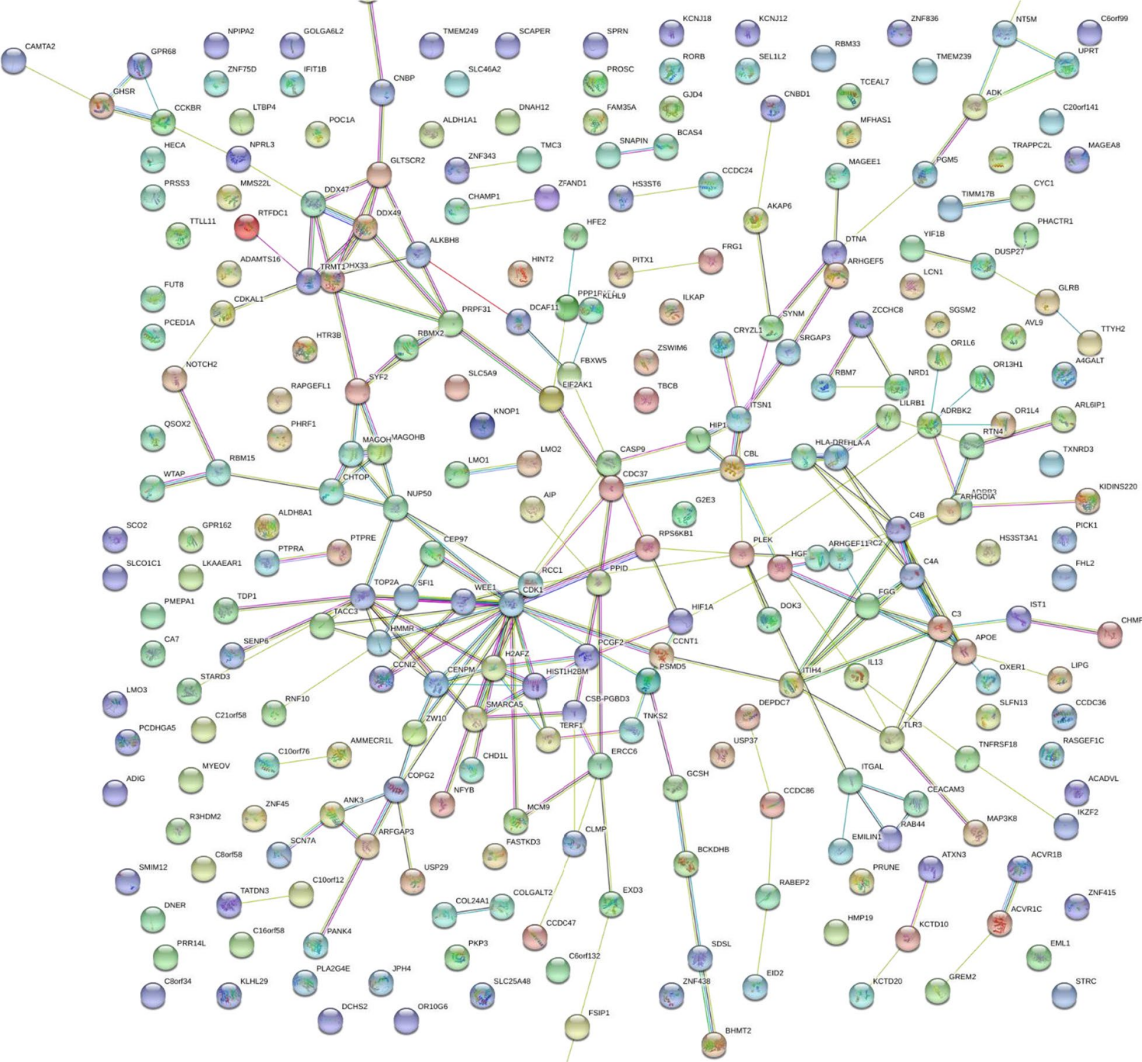
The breast cancer STRING network where Chi Square χ^2^ ≥ 15 from fully tryptic phospho peptides. Breast cancer TRYP STYP, frequency difference greater than 15 and χ^2^ value greater than 15 at degrees of freedom of 1 (p < 0.0001). Network Information: number of nodes, 191; number of edges, 182; average node degree, 1.91; avg. local clustering coefficient, 0.335; expected number of edges, 152; PPI enrichment p-value, 0.00911

The protein gene symbols with large Chi Square values were significantly enriched in proteins that showed a complex set of previously established functional and structural relationships by STRING analysis. In a computationally independent method to ensure the variation in proteins associated with breast cancer were not just the result of some random process, we analyzed the distribution of the known protein–protein interactions and the distribution of the cellular location, molecular function and biological processes of the proteins identified from endogenous peptides with respect to a random sampling of the human genome. There were many protein interactions apparent between the proteins computed to be specific to breast cancer from fully tryptic (Fig. 2) and/or phospho tryptic peptides (Fig. 3). The breast cancer samples showed statistically significant enrichment of protein interactions and Gene Ontology terms that were consistent with structural and functional relationships between the proteins identified in breast cancer compared to a random sampling of the human genome (Tables 2, 3, 4): STRING analysis of the breast cancer specific proteins detected by fully tryptic peptides and/or fully tryptic phosphopeptides with a Chi Square (χ^2^) value of ≥ 9 showed a significant protein interaction [Network Stats: number of nodes, 1580; number of edges, 9987; average node degree, 12.6; avg. local clustering coefficient, 0.272; expected number of edges, 8736; PPI enrichment p-value < 1.0e−16].

**Table 2:** STRING analysis of Biological Process of Gene Symbol distributions from the TRYP and TRYP STYP where delta and χ^2^ were both greater than 9 after correction

#Term ID	Term description	Observed gene count	Background gene count	False discovery rate
O:0016043	Cellular component organization	551	5163	4.00E−09
O:0071840	Cellular component organization or biogenesis	567	5342	4.00E−09
O:0007017	Microtubule-based process	106	605	1.17E−08
O:0051641	Cellular localization	267	2180	7.59E−08
O:0006996	Organelle organization	356	3131	8.96E−08
O:0007010	Cytoskeleton organization	139	953	3.36E−07
O:0007018	Microtubule-based movement	57	276	3.34E−06
O:0007399	Nervous system development	257	2206	7.50E−06
O:0008104	Protein localization	230	1966	3.52E−05
O:0120036	Plasma membrane bounded cell projection organization	138	1034	3.52E−05
O:0048731	System development	427	4144	4.68E−05
O:0033036	Macromolecule localization	257	2268	4.69E−05
O:0070727	Cellular macromolecule localization	171	1374	4.83E−05
O:0030030	Cell projection organization	140	1067	4.96E−05
O:0034613	Cellular protein localization	169	1367	7.45E−05
O:0009987	Cellular process	1271	14652	0.00013
O:0051179	Localization	516	5233	0.00015
O:0043170	Macromolecule metabolic process	702	7453	0.00018
O:0007275	Multicellular organism development	470	4726	0.00025
O:0032502	Developmental process	528	5401	0.00025
O:0051649	Establishment of localization in cell	189	1616	0.0003
O:0046907	Intracellular transport	167	1390	0.00031
O:0090304	Nucleic acid metabolic process	399	3941	0.00043
O:0051128	Regulation of cellular component organization	252	2306	0.00047
O:0007156	Homophilic cell adhesion via plasma membrane adhesion molecules	34	158	0.00063
O:0048856	Anatomical structure development	496	5085	0.00066
O:0006139	Nucleobase-containing compound metabolic process	449	4551	0.00082
O:0007155	Cell adhesion	110	843	0.00082
O:0006928	Movement of cell or subcellular component	160	1355	0.001
O:0051276	Chromosome organization	125	999	0.001
O:0097435	Supramolecular fiber organization	60	383	0.0012
O:0046483	Heterocycle metabolic process	459	4716	0.002
O:0048666	Neuron development	99	758	0.002
O:0000226	Microtubule cytoskeleton organization	60	393	0.0022
O:0019219	Regulation of nucleobase-containing compound metabolic process	408	4133	0.0022
O:0044260	Cellular macromolecule metabolic process	602	6413	0.0022
O:0051130	Positive regulation of cellular component organization	135	1128	0.0025
O:0006725	Cellular aromatic compound metabolic process	460	4754	0.0028
O:0060255	Regulation of macromolecule metabolic process	572	6072	0.0028
O:0098609	Cell–cell adhesion	62	416	0.0028
O:0044085	Cellular component biogenesis	267	2556	0.0029
O:0051252	Regulation of RNA metabolic process	385	3890	0.0029
O:0010468	Regulation of gene expression	440	4533	0.0033
O:0022607	Cellular component assembly	247	2343	0.0034
O:0048699	Generation of neurons	162	1422	0.0034
O:0071166	Ribonucleoprotein complex localization	27	125	0.0034
O:0030182	Neuron differentiation	115	940	0.0038
O:0032989	Cellular component morphogenesis	93	720	0.0038
O:0098742	Cell–cell adhesion via plasma-membrane adhesion molecules	40	230	0.0038
O:0031175	Neuron projection development	82	616	0.0043
O:0006611	Protein export from nucleus	29	144	0.0048
O:0016070	RNA metabolic process	342	3430	0.0048
O:0031323	Regulation of cellular metabolic process	569	6082	0.0048
O:0050794	Regulation of cellular process	929	10484	0.0048
O:1901360	Organic cyclic compound metabolic process	474	4963	0.0049
O:0051168	Nuclear export	31	161	0.005
O:0080090	Regulation of primary metabolic process	560	5982	0.005
O:0051640	Organelle localization	77	574	0.0051
O:0006403	RNA localization	37	211	0.0053
O:0019222	Regulation of metabolic process	604	6516	0.0053
O:0035023	Regulation of Rho protein signal transduction	27	131	0.0053
O:2000112	Regulation of cellular macromolecule biosynthetic process	395	4050	0.0053
O:0000902	Cell morphogenesis	82	626	0.0054
O:0051171	Regulation of nitrogen compound metabolic process	546	5827	0.0054
O:0071426	Ribonucleoprotein complex export from nucleus	26	124	0.0054
O:0033043	Regulation of organelle organization	134	1155	0.0058
O:0048468	Cell development	166	1493	0.0058
O:0050658	RNA transport	34	189	0.006
O:0006355	Regulation of transcription, DNA-templated	360	3661	0.0061
O:0006405	RNA export from nucleus	27	134	0.0061
O:0010467	Gene expression	366	3733	0.0061
O:0022008	Neurogenesis	168	1519	0.0061
O:0051056	Regulation of small TPase mediated signal transduction	48	310	0.0061
O:0065007	Biological regulation	1026	11740	0.0061
O:0003205	Cardiac chamber development	31	166	0.0062
O:1903506	Regulation of nucleic acid-templated transcription	361	3683	0.0068
O:0010556	Regulation of macromolecule biosynthetic process	400	4143	0.0079
O:0006406	mRNA export from nucleus	23	107	0.0083
O:0015833	Peptide transport	157	1416	0.0084
O:0032501	Multicellular organismal process	599	6507	0.0092
O:0051493	Regulation of cytoskeleton organization	65	477	0.0092

The protein–protein interaction statistics were: 485 nodes; 1148 edges; average node degree, 4.73; avg. local clustering coefficient, 0.325; expected number of edges: 851; PPI enrichment p-value: < 1.0e−16

**Table 3:** STRING analysis of Molecular Function of Gene Symbol distributions from the TRYP and TRYP STYP where delta and χ^2^ were both greater than 9 after correction

#Term ID	Term description	Observed gene count	Background gene count	False discovery rate
GO:0005488	Binding	1152	11878	9.77E−20
GO:0005515	Protein binding	694	6605	3.83E−13
GO:0005524	ATP binding	209	1462	1.30E−11
GO:0043167	Ion binding	637	6066	1.30E−11
GO:0032559	Adenyl ribonucleotide binding	213	1514	1.80E−11
GO:0008144	Drug binding	227	1710	3.62E−10
GO:0035639	Purine ribonucleoside triphosphate binding	232	1794	1.81E−09
GO:0032553	Ribonucleotide binding	238	1868	3.13E−09
GO:0032555	Purine ribonucleotide binding	236	1853	3.33E−09
GO:0097159	Organic cyclic compound binding	560	5382	3.33E−09
GO:1901363	Heterocyclic compound binding	552	5305	4.36E−09
GO:0097367	Carbohydrate derivative binding	265	2163	4.89E−09
GO:0000166	Nucleotide binding	258	2097	5.95E−09
GO:0008092	Cytoskeletal protein binding	130	882	5.00E−08
GO:0003779	Actin binding	76	413	6.27E−08
GO:0043168	Anion binding	309	2696	6.27E−08
GO:0016887	ATPase activity	73	392	7.90E−08
GO:0036094	Small molecule binding	282	2460	3.63E−07
GO:0042623	ATPase activity, coupled	60	320	1.76E−06
GO:0017111	Nucleoside-triphosphatase activity	111	778	3.30E−06
GO:0004386	Helicase activity	36	147	4.31E−06
GO:0016462	Pyrophosphatase activity	114	819	6.34E−06
GO:0046872	Metal ion binding	420	4087	6.91E−06
GO:0043169	Cation binding	425	4170	1.22E−05
GO:0003777	Microtubule motor activity	29	110	1.74E−05
GO:0008017	Microtubule binding	48	253	2.25E−05
GO:0051015	Actin filament binding	35	158	3.76E−05
GO:0019899	Enzyme binding	241	2197	9.02E−05
GO:0003774	Motor activity	30	131	0.00012
GO:0015631	Tubulin binding	55	344	0.00032
GO:0051020	GTPase binding	83	614	0.00064
GO:0017048	Rho GTPase binding	32	162	0.00073
GO:0003682	Chromatin binding	69	501	0.0018
GO:0005089	Rho guanyl-nucleotide exchange factor activity	19	76	0.0025
GO:0003676	Nucleic acid binding	330	3332	0.0028
GO:0005198	Structural molecule activity	86	679	0.0032
GO:0031267	Small GTPase binding	70	525	0.0036
GO:0004672	Protein kinase activity	81	635	0.0039
GO:0140096	Catalytic activity, acting on a protein	225	2176	0.005
GO:0019904	Protein domain specific binding	87	706	0.0061
GO:0005085	Guanyl-nucleotide exchange factor activity	46	311	0.0066
GO:0005509	Calcium ion binding	86	700	0.007
GO:0017016	Ras GTPase binding	66	510	0.0103
GO:0005516	Calmodulin binding	32	194	0.0106
GO:0004674	Protein serine/threonine kinase activity	59	444	0.011
GO:0051010	Microtubule plus-end binding	7	13	0.0119
GO:0005088	Ras guanyl-nucleotide exchange factor activity	37	243	0.0143
GO:0005096	GTPase activator activity	40	278	0.023
GO:0004004	ATP-dependent RNA helicase activity	15	66	0.0237
GO:0016773	Phosphotransferase activity, alcohol group as acceptor	89	767	0.0237
GO:0030695	GTPase regulator activity	43	307	0.0237
GO:0060589	Nucleoside-triphosphatase regulator activity	47	345	0.0237
GO:0044877	Protein-containing complex binding	108	968	0.0241
GO:0016772	Transferase activity, transferring phosphorus-containing groups	109	982	0.0255
GO:0032947	Protein-containing complex scaffold activity	15	68	0.0267
GO:0008047	Enzyme activator activity	63	510	0.0303
GO:0097493	Structural molecule activity conferring elasticity	7	17	0.0325
GO:0016301	Kinase activity	94	835	0.0352
GO:0051959	Dynein light intermediate chain binding	9	29	0.0352
GO:0042800	Histone methyltransferase activity (H3-K4 specific)	5	8	0.0388
GO:0008094	DNA-dependent ATPase activity	14	66	0.0482
GO:0140030	Modification-dependent protein binding	22	131	0.0482
GO:0008026	ATP-dependent helicase activity	17	90	0.0499

Additional details see Table 2

**Table 4:** STRING analysis of cellular component of Gene Symbol distribution from the TRYP and TRYP STYP where delta and χ^2^ were both greater than 9 after correction

#Term ID	Term description	Observed gene count	Background gene count	False discovery rate
GO:0005622	Intracellular	1302	14286	1.22E−14
GO:0044424	Intracellular part	1282	13,996	1.22E−14
GO:0005856	Cytoskeleton	281	2068	4.49E−14
GO:0043232	Intracellular non-membrane-bounded organelle	467	4005	4.49E−14
GO:0044464	Cell part	1417	16,244	4.88E−11
GO:0043226	Organelle	1143	12,432	7.73E−11
GO:0043229	Intracellular organelle	1124	12,193	9.50E−11
GO:0044430	Cytoskeletal part	207	1547	1.04E−09
GO:0032991	Protein-containing complex	501	4792	2.68E−08
GO:0042995	Cell projection	242	1969	2.68E−08
GO:0044422	Organelle part	862	9111	4.13E−08
GO:0120025	Plasma membrane bounded cell projection	234	1900	4.13E−08
GO:0005737	Cytoplasm	1030	11,238	5.39E−08
GO:0005634	Nucleus	676	6892	9.10E−08
GO:0044428	Nuclear part	455	4359	2.36E−07
GO:0031981	Nuclear lumen	425	4030	2.95E−07
GO:0015630	Microtubule cytoskeleton	150	1118	4.50E−07
GO:0044446	Intracellular organelle part	834	8882	4.50E−07
GO:0044451	Nucleoplasm part	145	1073	4.89E−07
GO:0043005	Neuron projection	149	1142	2.14E−06
GO:0099081	Supramolecular polymer	122	880	2.14E−06
GO:0070013	Intracellular organelle lumen	516	5162	2.48E−06
GO:0120038	Plasma membrane bounded cell projection part	165	1316	3.34E−06
GO:0099568	Cytoplasmic region	68	402	3.64E−06
GO:0099512	Supramolecular fiber	118	873	8.36E−06
GO:0030054	Cell junction	131	1006	1.11E−05
GO:0043227	Membrane-bounded organelle	1007	11,244	1.79E−05
GO:0005930	Axoneme	28	107	1.90E−05
GO:0005654	Nucleoplasm	357	3446	2.24E−05
GO:0043231	Intracellular membrane-bounded organelle	936	10,365	2.24E−05
GO:0044420	Extracellular matrix component	20	59	2.55E−05
GO:0097458	Neuron part	171	1449	4.59E−05
GO:0005829	Cytosol	485	4958	5.91E−05
GO:0032838	Plasma membrane bounded cell projection cytoplasm	36	179	9.90E−05
GO:0098644	Complex of collagen trimmers	11	19	0.00014
GO:0015629	Actin cytoskeleton	65	432	0.00016
GO:0030424	Axon	75	530	0.00023
GO:0030016	Myofibril	39	216	0.00034
GO:0005911	Cell–cell junction	60	402	0.00042
GO:0043292	Contractile fiber	40	228	0.00045
GO:0062023	Collagen-containing extracellular matrix	29	144	0.00069
GO:0016604	Nuclear body	94	742	0.00088
GO:0044449	Contractile fiber part	37	212	0.00093
GO:0031012	Extracellular matrix	45	283	0.0011
GO:0016459	Myosin complex	18	69	0.0012
GO:0031965	Nuclear membrane	46	300	0.0019
GO:0005874	Microtubule	55	385	0.0022
GO:0005581	Collagen trimer	20	88	0.0024
GO:0098862	Cluster of actin-based cell projections	27	143	0.0028
GO:0005815	Microtubule organizing center	85	683	0.0029
GO:0044444	Cytoplasmic part	832	9377	0.0029
GO:0044441	Ciliary part	58	421	0.0032
GO:0005583	Fibrillar collagen trimer	7	11	0.0033
GO:0016460	Myosin II complex	11	32	0.0039
GO:0033267	Axon part	49	341	0.0039
GO:0014704	Intercalated disc	14	51	0.0041
GO:0005859	Muscle myosin complex	10	27	0.0044
GO:0008023	Transcription elongation factor complex	14	52	0.0047
GO:0032982	Myosin filament	9	22	0.0047
GO:0034399	Nuclear periphery	25	134	0.0047
GO:0044291	Cell–cell contact zone	16	67	0.0055
GO:0005915	Zonula adherens	6	9	0.0069
GO:0005694	Chromosome	108	950	0.0076
GO:0005929	Cilium	71	570	0.0076
GO:0030496	Midbody	28	165	0.0076
GO:0043034	Costamere	8	19	0.008
GO:0044447	Axoneme part	10	31	0.0093
GO:0005913	Cell–cell adherens junction	16	72	0.0098
GO:0032420	Stereocilium	12	44	0.0098
GO:0005875	Microtubule associated complex	25	144	0.01
GO:0016607	Nuclear speck	51	381	0.01
GO:0031252	Cell leading edge	50	371	0.01
GO:0032421	Stereocilium bundle	13	51	0.01
GO:0033268	Node of Ranvier	7	15	0.01
GO:0097060	Synaptic membrane	43	308	0.0114
GO:0034708	Methyltransferase complex	18	90	0.0124
GO:0042383	Sarcolemma	22	122	0.0124
GO:0030056	Hemidesmosome	5	7	0.0137
GO:0098590	Plasma membrane region	116	1061	0.0141
GO:0044450	Microtubule organizing center part	27	167	0.0147
GO:0090543	Flemming body	9	28	0.0147
GO:0005814	Centriole	22	125	0.0152
GO:0030017	Sarcomere	30	195	0.0159
GO:0042405	Nuclear inclusion body	6	12	0.016
GO:0070161	Anchoring junction	38	270	0.0172
GO:0005635	Nuclear envelope	56	446	0.0183
GO:0036396	RNA N6-methyladenosine methyltransferase complex	5	8	0.019
GO:0005813	Centrosome	58	468	0.0194
GO:0005730	Nucleolus	102	926	0.0196
GO:0030427	Site of polarized growth	26	164	0.0203
GO:0045211	Postsynaptic membrane	34	237	0.0207
GO:0030018	Z disc	21	122	0.0217
GO:0098858	Actin-based cell projection	29	192	0.0217
GO:0016363	Nuclear matrix	19	106	0.0228
GO:0005938	Cell cortex	33	230	0.0229
GO:0030027	Lamellipodium	28	185	0.024
GO:0044304	Main axon	14	67	0.0242
GO:0070449	Elongin complex	5	9	0.0246
GO:0005604	Basement membrane	17	91	0.0248
GO:0043194	Axon initial segment	6	14	0.0248
GO:0005912	Adherens junction	35	252	0.0263
GO:0099513	Polymeric cytoskeletal fiber	73	645	0.0402
GO:0005587	Collagen type IV trimer	4	6	0.0406
GO:1990752	Microtubule end	7	22	0.0413
GO:0030426	Growth cone	24	159	0.0442
GO:0044427	Chromosomal part	89	819	0.0442
GO:0005858	Axonemal dynein complex	6	17	0.0499
GO:0035371	Microtubule plus-end	6	17	0.0499

Additional details see Table 2

### ANOVA analysis across disease, normal and control plasma treatments

**Fig. 4:**
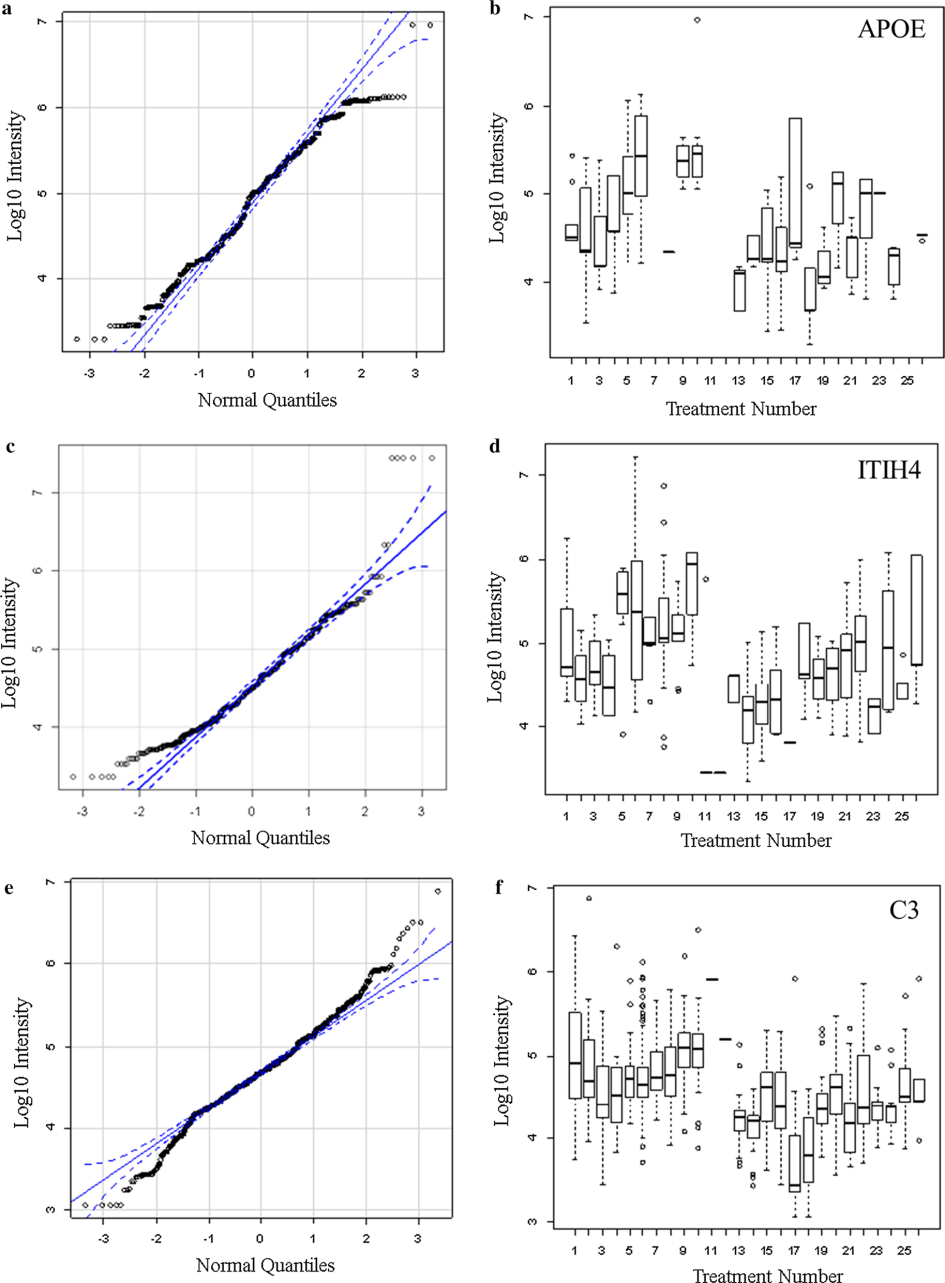
The distributions of log10 precursor intensity by quantile and quantile box plots of APOE, ITIH4, and C3 across the disease and control treatments.** a** APOE log_10_ peptide intensity quantile plot;** b** APOE log_10_ peptide intensity quantile box plot;** c** ITIH4 log_10_ peptide intensity quantile plot;** d** ITIH4 log_10_ peptide intensity quantile box plot;** e** C3 log10 peptide intensity quantile plot;** f** C3 log10 peptide intensity quantile box plot; Treatment ID numbers: 1, Alzheimer normal; 2, Alzheimer’s normal control STYP; 3, Alzheimer’s dementia; 4, Alzheimer’s dementia STYP; 5, Cancer breast; 6, Cancer breast STYP; 7, Cancer control; 8, Cancer control STYP; 9, Cancer ovarian; 10, Cancer ovarian STYP; 11, Ice Cold; 12, Ice Cold STYP; 13, Heart attack Arterial; 14 Heart attack Arterial STYP; 15, Heart attack normal control, 16, Heart attack normal Control STYP; 17, Heart attack; 18, Heart attack STYP; 19, Multiple Sclerosis normal control; 20, Multiple sclerosis normal control STYP; Multiple sclerosis; 22, Multiple Sclerosis STYP, 23 Sepsis; 24, Sepsis STYP; 25, Sepsis normal control; 26, Sepsis normal control STYP. There was significant effects of treatments and peptides by two-way ANOVA. Analysis of the proteins shown across treatments produced a significant F Statistic by one-way ANOVA. Note that many proteins were not detected in the ice cold plasma

**Fig. 5:**
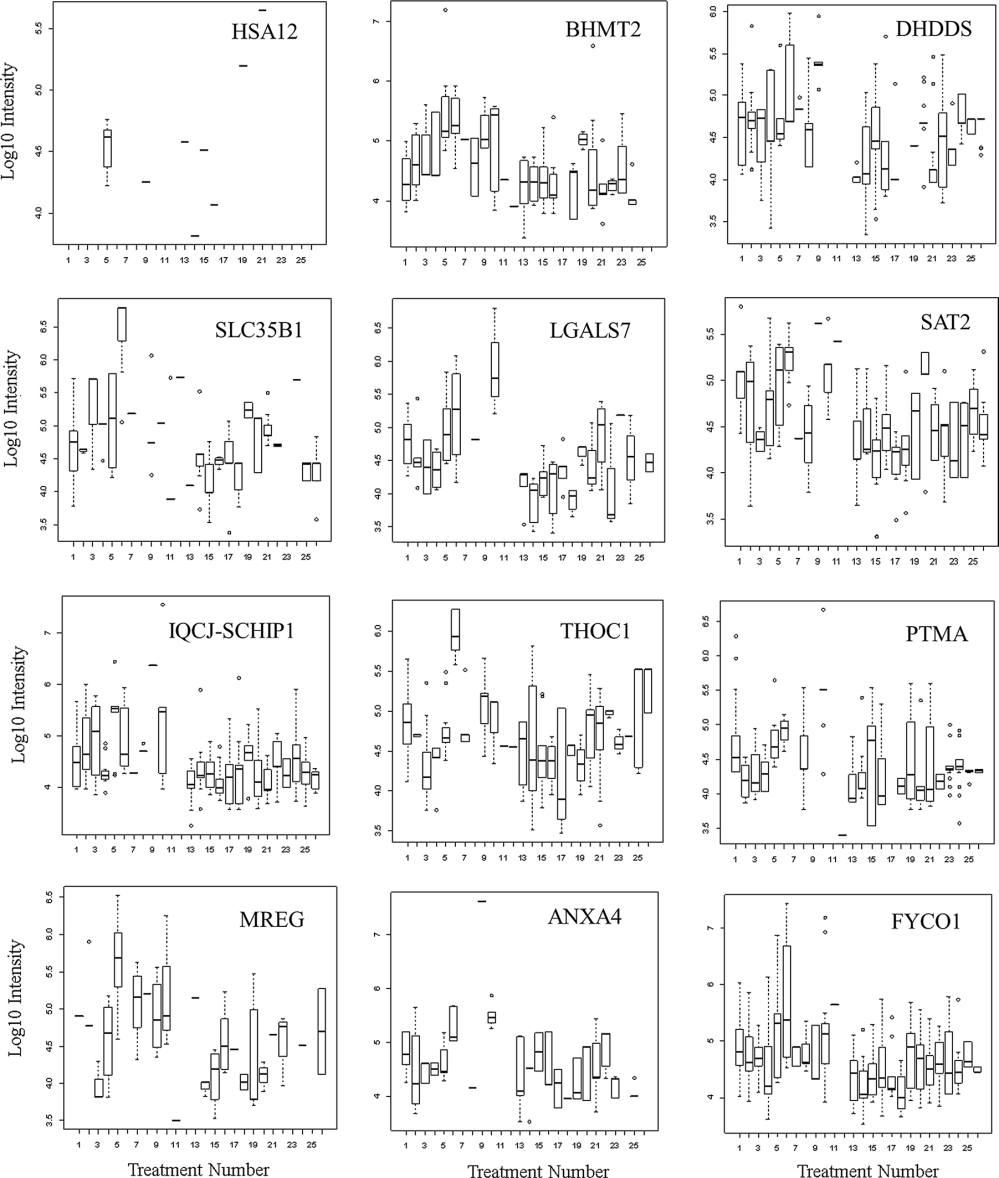
Quantile box plots showing the distribution of log10 precursor intensity by quantile box plots of HSA12, BHMT2, DHDDS, SLC35B1, LGALS7, SAT2, IQCJ-SCHIP1 fusion, THOC1, PTMA, MREG, ANXA4 and FYCO1 across the disease and control treatments. Box plots show log_10_ intensity versus treatment number for gene symbol indicated. Treatment ID numbers: 1, Alzheimer normal; 2, Alzheimer’s normal control STYP; 3, Alzheimer’s dementia; 4, Alzheimer’s dementia STYP; 5, Cancer breast; 6, Cancer breast STYP; 7, Cancer control; 8, Cancer control STYP; 9, Cancer ovarian; 10, Cancer ovarian STYP; 11, Ice Cold; 12, Ice Cold STYP; 13, Heart attack Arterial; 14 Heart attack Arterial STYP; 15, Heart attack normal control, 16, Heart attack normal Control STYP; 17, Heart attack; 18, Heart attack STYP; 19, Multiple Sclerosis normal control; 20, Multiple sclerosis normal control STYP; Multiple Sclerosis; 22, Multiple sclerosis STYP, 23 Sepsis; 24, Sepsis STYP; 25, Sepsis normal control; 26, Sepsis normal control STYP. There was significant effects of treatments and peptides by two-way ANOVA. Analysis of the proteins shown across treatments produced a significant F Statistic by one-way ANOVA. Note that many proteins were not detected in the ice cold plasma

**Fig. 6:**
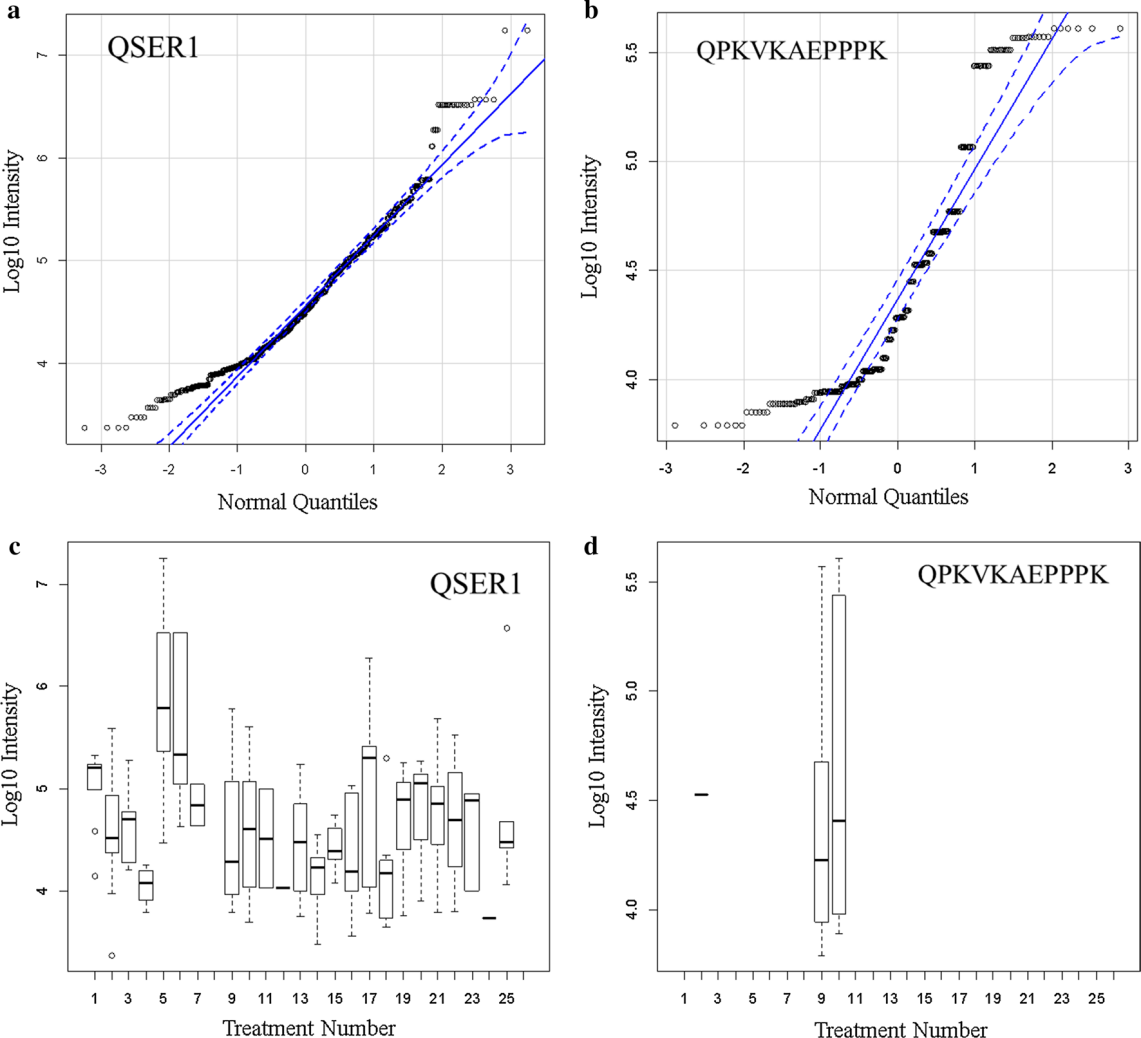
QSER1 ANOVA analysis and Tukey–Kramer HSD multiple means comparison of breast versus ovarian cancer and other diseases and normal treatments.** a** All QSER1 peptides quantile plot; **b** QSER1 peptide QPKVKAEPPPK quantile plot; **c** All QSER1 peptides box plot see ANOVA below; **d** QSER1 peptide QPKVKAEPPPK box plot. Treatment ID numbers: 1, Alzheimer normal; 2, Alzheimer’s normal control STYP; 3, Alzheimer’s dementia; 4, Alzheimer’s dementia STYP; 5, Cancer breast; 6, Cancer breast STYP; 7, Cancer control; 8, Cancer control STYP; 9, Cancer ovarian; 10, Cancer ovarian STYP; 11, Ice Cold; 12, Ice Cold STYP; 13, Heart attack Arterial; 14 Heart attack Arterial STYP; 15, Heart attack normal control, 16, Heart attack normal Control STYP; 17, Heart attack; 18, Heart attack STYP; 19, Multiple Sclerosis normal control; 20, Multiple Sclerosis normal control STYP; Multiple sclerosis; 22, Multiple sclerosis STYP, 23 Sepsis; 24, Sepsis STYP; 25, Sepsis normal control; 26, Sepsis normal control STYP. There was significant effects of treatments and peptides by two-way ANOVA (not shown). One way ANOVA:Df Sum Sq Mean Sq F value Pr(> F), Treatment_ID 23 113.0 4.912 16.55 < 2e−16 ***Residuals 808 239.9 0.297

Many proteins that showed greater observation frequency in breast cancer also showed significant variation in precursor intensity compared to ovarian cancer, the female normal controls and male or female EDTA plasma from other disease and normal plasma by ANOVA comparison. The mean precursor intensity values from gene symbols that varied by Chi Square (χ^2^ > 15) were subsequently analyzed by univariate ANOVA in R to look for proteins that showed differences in ion precursor intensity values across treatments [12, 16] (Figs. 4, 5, 6). Common plasma proteins including APOE, ITIH4 and C3 showed significantly different intensity between breast cancer versus ovarian cancer and normal plasma (Fig. 4). Analysis of the frequently observed proteins by quantile box plots and ANOVA confirmed increases in mean precursor intensity in cancer associated proteins as SLC35B1, IQCJ-SCHIP1, MREG, BHMT2, LGALS7, THOC1, ANXA4, DHDDS, SAT2, PTMA, FYCO1 and ZNF562 among others between breast cancer versus ovarian cancer and/or other disease or normal plasma (Fig. 5). HSA12 represents many proteins that were observed only in breast cancer but were apparently only sporadically detected and require further consideration. Glutamine Serine Rich Protein 1 (QSER1) was observed most frequently in ovarian cancer (Table 5). In contrast, QSER1 showed higher average intensity in breast cancer than ovarian cancer or any other disease and normal by ANOVA followed by the Tukey–Kramer HSD test (Fig. 6) when all peptides were considered. However, the peptide QPKVKAEPPPK, that was specific to QSER1 by BLAST [62], was observed in ovarian cancer but was not observed in other samples (Fig. 6d).

**Table 5:** The analysis of mean peptide intensity per gene symbol for QSER1 protein by ANOVA with Tukey–Kramer multiple means comparison

Treatment	Mean	SD	Data N	Tukey–Kramer
1	5.072769	0.302986	21	d
2	4.593409	0.511989	67	cde
3	4.633497	0.3285	26	bde
4	4.056312	0.161037	33	a
5	5.918212	0.760851	25	h
6	5.717592	0.763346	18	h
7	4.837276	0.216573	8	bdef
9	4.542693	0.65645	141	ceg
10	4.600209	0.640097	66	cde
11	4.512103	0.515631	8	acde
12	4.029774	0	4	acde
13	4.452935	0.491664	50	aceg
14	4.12479	0.351469	35	af
15	4.419355	0.198763	53	ace
16	4.324212	0.504538	32	ace
17	4.928881	0.947319	22	dg
18	4.173403	0.478339	36	ab
19	4.740343	0.428142	58	cde
20	4.80151	0.475907	35	de
21	4.749583	0.513686	36	cde
22	4.755553	0.517117	25	cde
23	4.58392	0.466147	11	acde
24	3.736293	0	4	abc
25	4.881761	0.953098	18	de

Treatment ID numbers: 1, Alzheimer normal; 2, Alzheimer’s normal control STYP; 3, Alzheimer’s dementia; 4, Alzheimer’s dementia STYP; 5, Cancer breast; 6, Cancer breast STYP; 7, Cancer control; 8, Cancer control STYP; 9, Cancer ovarian; 10, Cancer ovarian STYP; 11, Ice Cold; 12, Ice Cold STYP; 13, Heart attack Arterial; 14 Heart attack Arterial STYP; 15, Heart attack normal control, 16, Heart attack normal Control STYP; 17, Heart attack; 18, Heart attack STYP; 19, Multiple Sclerosis normal control; 20, Multiple sclerosis normal control STYP; Multiple sclerosis; 22, Multiple Sclerosis STYP, 23 Sepsis; 24, Sepsis STYP; 25, Sepsis normal control; 26, Sepsis normal control STYP. The Tukey–Kramer multiple comparison ranking of mean intensity from R is shown by letters

## Discussion

A simple and direct strategy to discover breast cancer-specific variation may be to compare plasma peptides and proteins to ovarian cancer and other disease and control sample sets under identical conditions. The aim and objective of this study was proof of concept towards a method to compare the endogenous trytic peptides of breast cancer plasma to those from multiple clinical treatments and locations that utilized random and independent sampling by a battery of robust and sensitive linear quadrupole ion traps where the results were compiled using the standard SQL Server and R statistical systems. Random and independent sampling of peptides from step-wise fractionation followed by LC–ESI–MS/MS is a time and manual labor intensive approach that is sensitive, direct, and rests on few assumptions [17, 38]. High signal to noise ratio of blood peptides is dependent on sample preparation to break the sample into many sub-fractions to relieve competition and suppression of ionization and thus achieve sensitivity [13, 21, 22] but then requires large computing power to re-assemble the sub-fractions, samples and treatments [14, 21, 38]. The careful study of pre-clinical variation over time, and under various storage and preservation conditions, seems to rule out pre-clinical variation as the most important source of variation between breast cancer and other disease and control treatments [17–19]. Together the results amount to a successful proof of principal for the application of random and independent sampling of plasma from multiple clinical locations by LC–ESI–MS/MS to identify and quantify proteins and peptides that show variation between sample populations. The approach shows great sensitivity and flexibility but relies on the fit of MS/MS spectra to assign peptide identity and statistical analysis of precursor ion counts and intensity by Chi Square and ANOVA and so is computationally intensive.

### Chi Square analysis of breast cancer versus ovarian cancer

The SQL Server and R statistical system permits the rapid statistical and graphical analysis of the data at the level of Gene symbols, proteins or peptides. The large differences in observation frequency between breast and ovarian cancer using Chi Square after correction by the number of mass spectra collected was a simple means to reveal proteins that may vary in expression between the related disease states. Examining the observation frequency across all twelve disease and control clinical sample sets was a direct means to look for Gene Symbols that showed greater frequency in one sample set such QSER1 or to look for its peptide QPKVKAEPPPK that was highly specific to ovarian cancer [39].

### Pathway and gene ontology analysis by the STRING algorithm

The set of breast cancer gene symbols that were significant from Chi Square analysis of the peptide frequency counts were independently confirmed by STRING analysis. The network analysis by STRING indicated that the peptides and proteins detected were not merely a random selection of the proteins from the human genome but showed statistically significant protein–protein interactions, and enrichment of specific cellular components, biological processes, and molecular functions associated with the biology of cancer. The significant results from STRING analysis indicated that the results could not have resulted from random sampling error between breast versus ovarian cancer. The previously established structural or functional relationships observed among the breast cancer specific gene symbols filtered by χ^2^ were consistent with the detection of bone fide variation between breast versus ovarian cancer. The STRING results apparently indicated that specific cellular protein complexes are released into the circulation of breast cancer patients [50]. The enrichment of proteins associated with cell polarity, cytoskeleton, plasma membrane bounded cell projection, microtubule cytoskeleton, supramolecular fiber and membrane-bounded organelle were all consistent with the activation of phagocytic functions in motile cancer cells.

### Breast versus ovarian cancer specific variation by ANOVA

ANOVA may be an independent means to confirm the results of frequency analysis. However, the interpretation of mean precursor intensity data by ANOVA [12] and the use of the Tukey–Kramer multiple comparison [15, 16] may be confounded by the different peptide sequences within each protein [32]. Specific endogenous tryptic peptides, were detected from breast cancer versus the corresponding ovarian cancer or the other disease and normal plasma after filtering proteins by Chi Square and ANOVA. When all peptides were considered, QSER1 showed significantly higher mean intensity in breast cancer but the QSER1 peptide QPKVKAEPPPK was observed more frequently in ovarian cancer. The exclusive observation of the peptide QPKVKAEPPPK in ovarian cancer samples seemed to indicate the presence or activation of a tryptic protease with a different selectivity for QSER1. An automated examination at the level of peptides and proteins may be required that is an even larger computational challenge. It should be possible to specifically compare and confirm the disease specific expression peptides and parent proteins by automatic targeted proteomics [18] after extraction of peptides [25] or after collection of the parent protein over the best partition chromatography resin [22] followed by tryptic digestion and analysis to test the discovery from this small experiment on a larger set of samples. For example, C4B peptides discovered by random and independent sampling were shown to be a marker of sample degradation by automatic targeted assays [17–19]. Automatic targeted analysis of peptides from independent analysis provided relative quantification to rapidly confirm the potential utility of C4B peptide as a marker of sample degradation [18]. Subsequently, the best performing peptides and proteins may be absolutely quantified by external or internal-isotopic standards to provide absolute quantification.

### Agreement with previous genetic and biochemical experiments

The striking agreement between the peptides and proteins observed in the plasma of breast cancer patients and the previous literature on breast cancer tumors, adjacent fluids, cell lines or blood fluids indicates that LC–ESI–MS/MS of blood peptides will be a powerful tool for selecting plasma proteins and peptides for further research and confirmation. The results of mass spectrometry show striking agreement with previous genetic or biochemical experiments on cancer tissues, tumors, biopsies or cell lines: CPEB1 [63], LTBP4 [64], HIF1A [65, 66], IGHE [67], RAB44 [68], NEFM [39], C19orf82, SLC35B1 [69], 1D12A that shows a cyptic alignment with cyclin-dependent kinase-like isoform 1 [70], C8orf34 [71], OCLN [72], EYA1 [73], HLA-DRB1 [74], LAR [75] and LRRC4B that interacts with the LARS receptor phosphatases [76], PTPDC1 [77], WWC1 [78], ZNF562, PTMA [79], MGAT1 [80], NDUFA1 [81], NOGOC [82], olfactory receptors OR1E or the HSA12 protein [83], GCSH [84], ELTD1 [85], TBX15 [86], orphan nuclear receptors such as NR2C2 [87], autophagy related proteins such as ATG16L1 (FLJ00045) that regulate the production of extracellular vesicles called exosomes [88], PDLIM1 [89, 90], GALNT9 [91], ASH2L [92], PPFIBP1 [93], SLCO3A1 [94], BHMT2 [95], CS citrate synthase [96] FAM188B2 inactive ubiquitin carboxyl-terminal hydrolase MINDY4B that is expressed in breast cancer tissue, LGALS7 [97] SAT2 [98], SFRS8, SLC22A12 [99], WNT9B [100], SLC2A4 [101], ZNF101, WT1 (Wilms Tumor Protein) [102], CCDC47 [103], ERLIN1 (SPFH1) and MREG [104], EID2 [105], THOC1 [106, 107], DDX47 [108], PTPRE [109], EMILIN1 [110], DKFZp779G1236 (piccolo, or piBRCA2) [111], MAP3K8 [112] regulated by Serine/Arginine-Rich Splicing Factor Kinase [113], QSER1 [39], IQCJ-SCHIP1 [114, 115], ANXA4 [116] and DHDDS [117] among others. The disease-specific proteins and peptides may result from the introduction of new proteins into circulation, or the release/activation of proteases in circulation, as a result of disease. The striking agreement of the plasma proteins observed here with the previous genomic, RNA expression and proteomic experiments on cancer tumors, fluids and cells indicates that comparing many and disease and control plasma samples by random and independent sampling with LC–ESI–MS/MS may be a direct and practical means to look for selective diagnostic and prognostic markers.

## Conclusion

The results of the step-wise organic extraction of peptides [21] provided for the enrichment of endogenous tryptic peptides with high signal to noise for random sampling [18] across disease and normal treatments. A large amount of proteomic data from multiple diseases, controls and institutions may be collected by random and independent sampling with a battery of robust and sensitive linear quadrupole ion traps and the results stored, related and statistically analyzed in 64 bit SQL Server/R. The LC–ESI–MS/MS of plasma endogenous tryptic peptides identified many blood proteins elevated in breast cancer that were previously associated with the biology of cancer or that have been shown to be biomarkers of solid tumors by genetic or biochemical methods. The striking level of agreement between the results of random and independent sampling of plasma by mass spectrometry with those from cancer tissues, fluids or cells indicated that clinical discovery of plasma by LC–ESI–MS/MS will be a powerful tool for clinical research. Peptide or proteins discovered by random and independent sampling of test samples might be confirmed by automatic targeted LC–ESI–MS/MS [17–19] from a larger cohort of independent samples. It was possible to discover peptides and/or proteins specific to breast cancer versus ovarian cancer and other diseases or normal plasma samples from many institutions using simple and disposable sample preparation, common instrumentation from the fit of MS/MS spectra using simple cross correlation or goodness of fit for storage with standard SQL database and classical statistical analysis with generic software.

## Supplementary information

**Additional file 1: Table S1.** Breast versus ovarian MSMS TRYP and STYP where both X2 where the corrected delta frequency is greater than 9.

## Data Availability

The raw data is provided in companion publication and the supplemental data.

## Abbreviations

TRYP: fully tryptic
TRYP STYP: fully tryptic and/or S, T or Y tryptic phosphopeptide
